# Supplementary material for: Structural basis of HLX10 PD-1 receptor recognition, a promising anti-PD-1 antibody clinical candidate for cancer immunotherapy
Source: PLoS One. 2021 Dec 31;16(12):e0257972. doi: 10.1371/journal.pone.0257972 (PMC8719770; doi:10.1371/journal.pone.0257972)
Supplement: S1 Fig — a. BLI binding kinetics. b. SPR binding kinetics of HLX10. c. SPR binding kinetics of Nivolumab. (PPTX) [file pone.0257972.s001.pptx]

## Slide 1
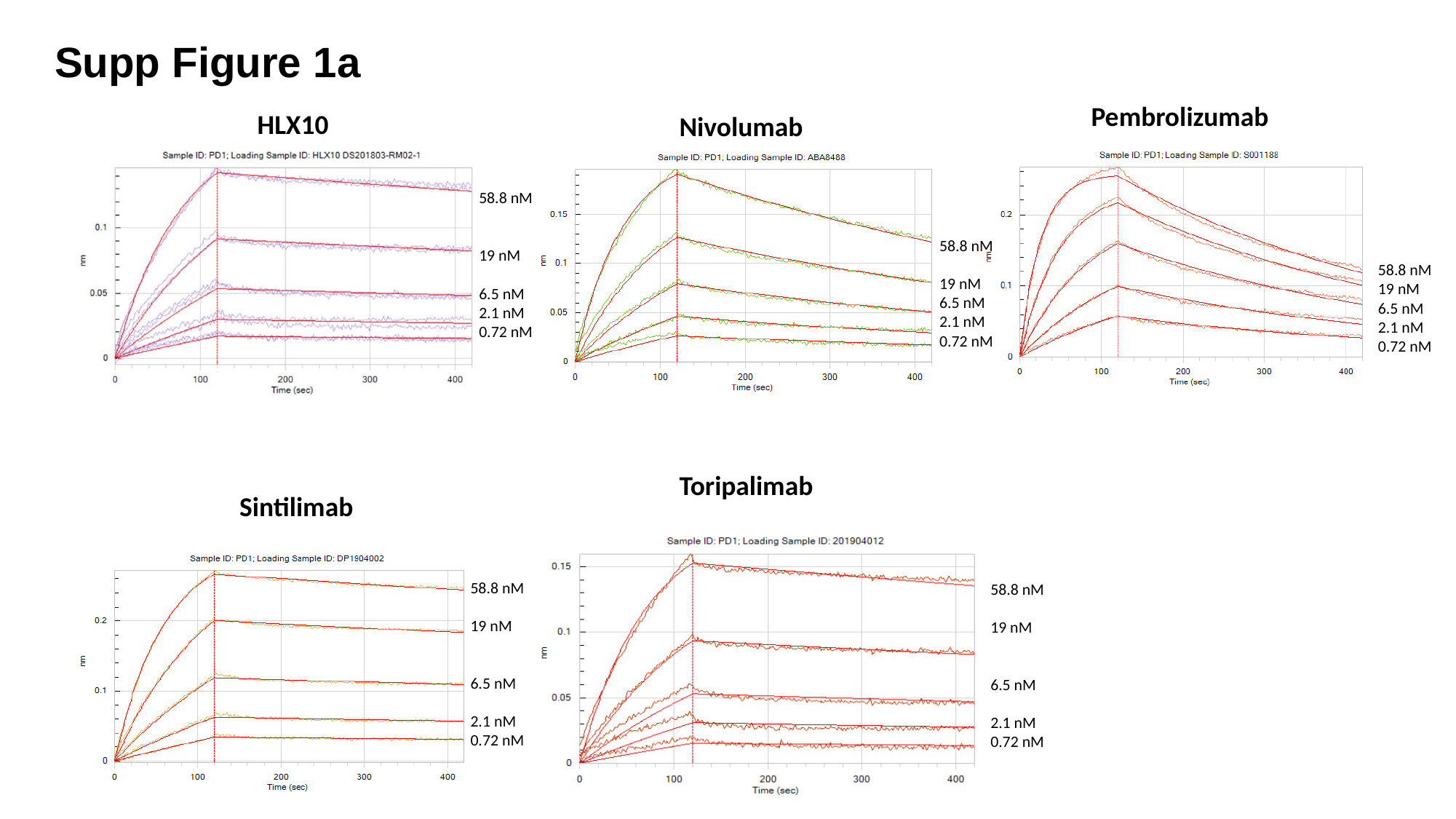

Supp Figure 1a
Pembrolizumab
HLX10
Nivolumab
58.8 nM
19 nM
6.5 nM
2.1 nM
0.72 nM
58.8 nM
19 nM
6.5 nM
2.1 nM
0.72 nM
58.8 nM
19 nM
6.5 nM
2.1 nM
0.72 nM
Toripalimab
Sintilimab
58.8 nM
19 nM
6.5 nM
2.1 nM
0.72 nM
58.8 nM
19 nM
6.5 nM
2.1 nM
0.72 nM

## Slide 2
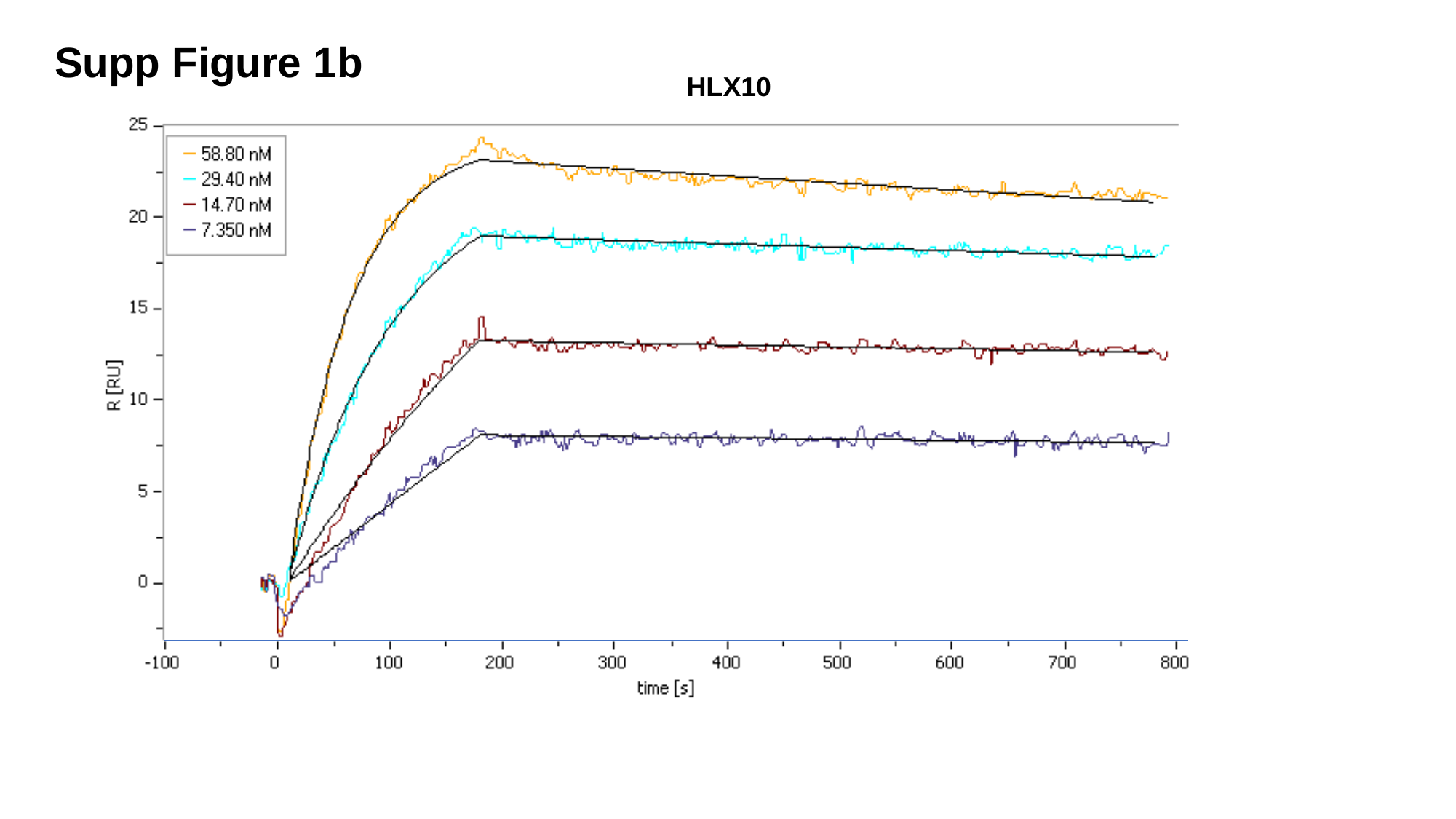

Supp Figure 1b
HLX10

## Slide 3
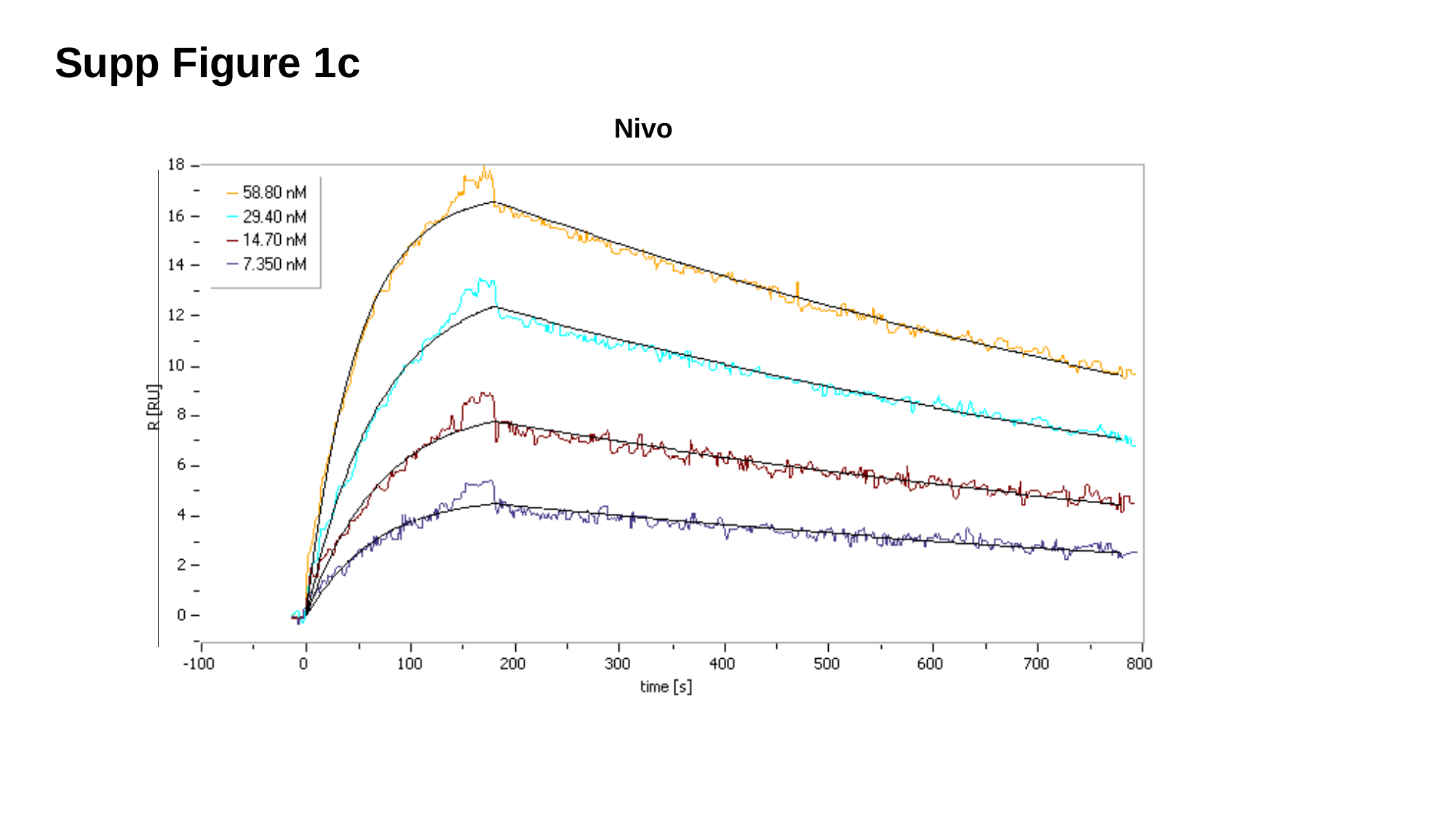

Supp Figure 1c
Nivo
