# Supplementary figures and images for: Structural basis of HLX10 PD-1 receptor recognition, a promising anti-PD-1 antibody clinical candidate for cancer immunotherapy
Source: PLoS One. 2021 Dec 31;16(12):e0257972. doi: 10.1371/journal.pone.0257972 (PMC8719770; doi:10.1371/journal.pone.0257972)

## Slide 1
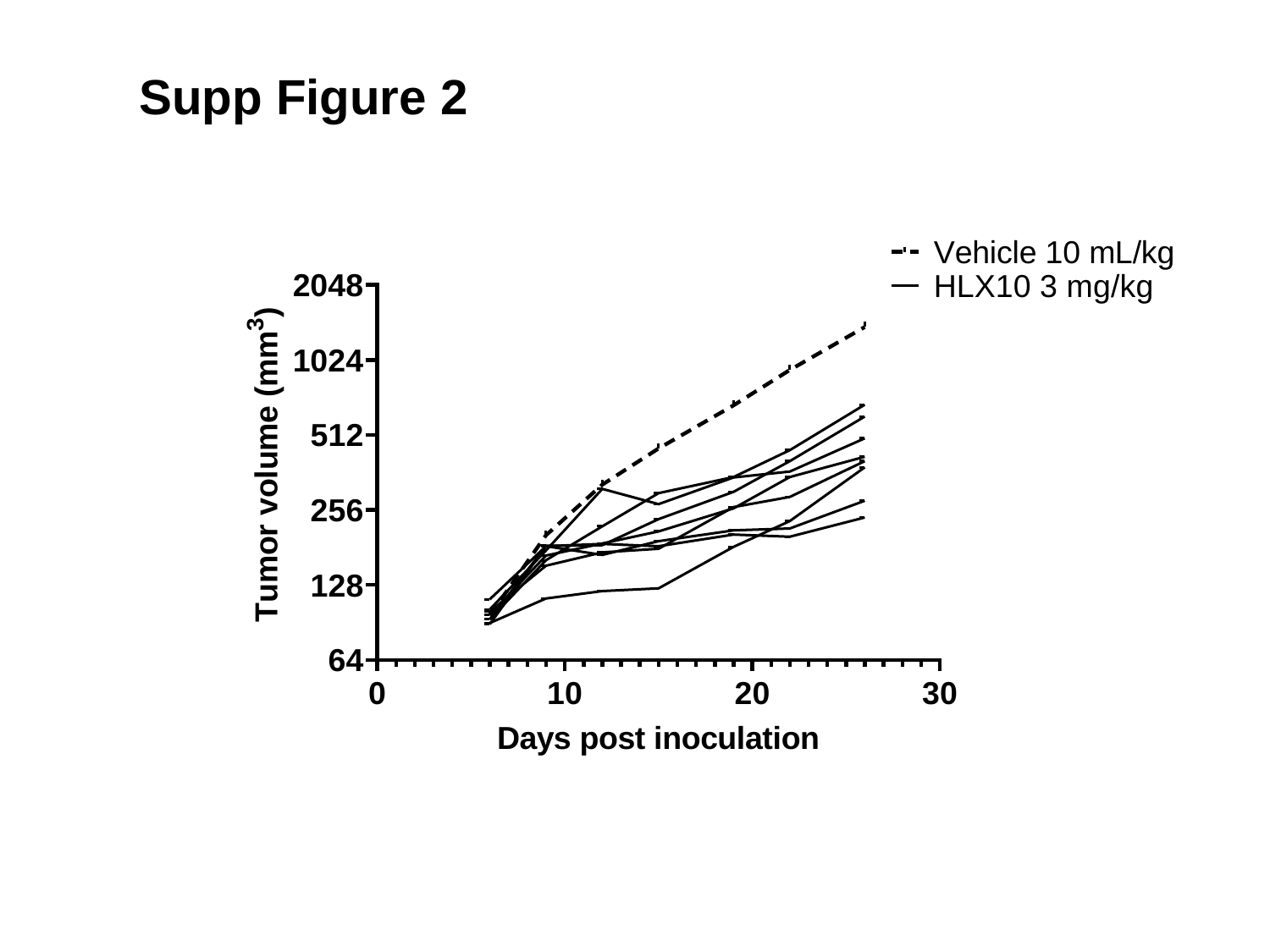

Supp Figure 2

Supplement: S2 Fig — (PPTX) [file pone.0257972.s002.pptx]

## Slide 1
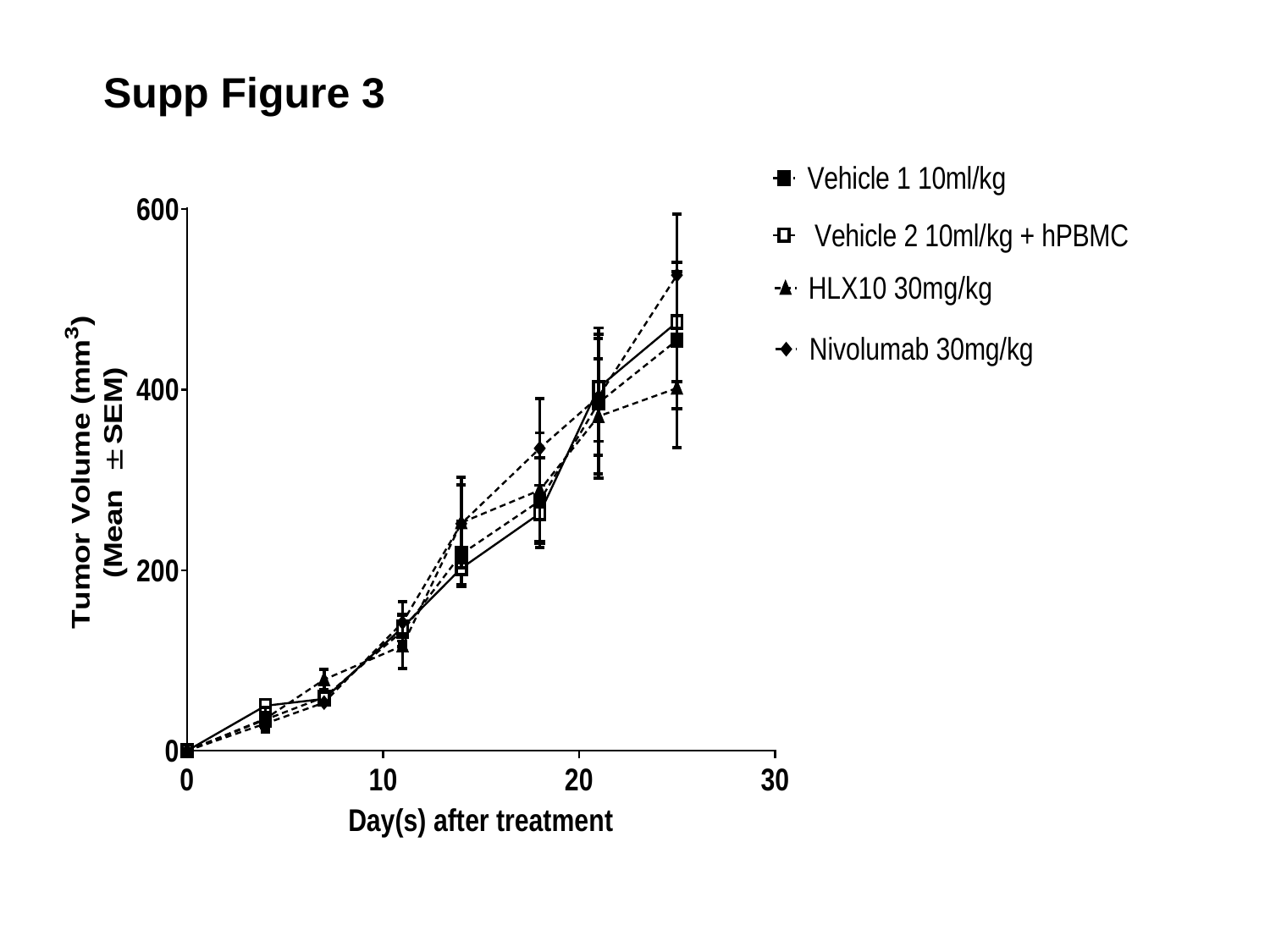

Supp Figure 3

Supplement: S3 Fig — (PPTX) [file pone.0257972.s003.pptx]

## Slide 1
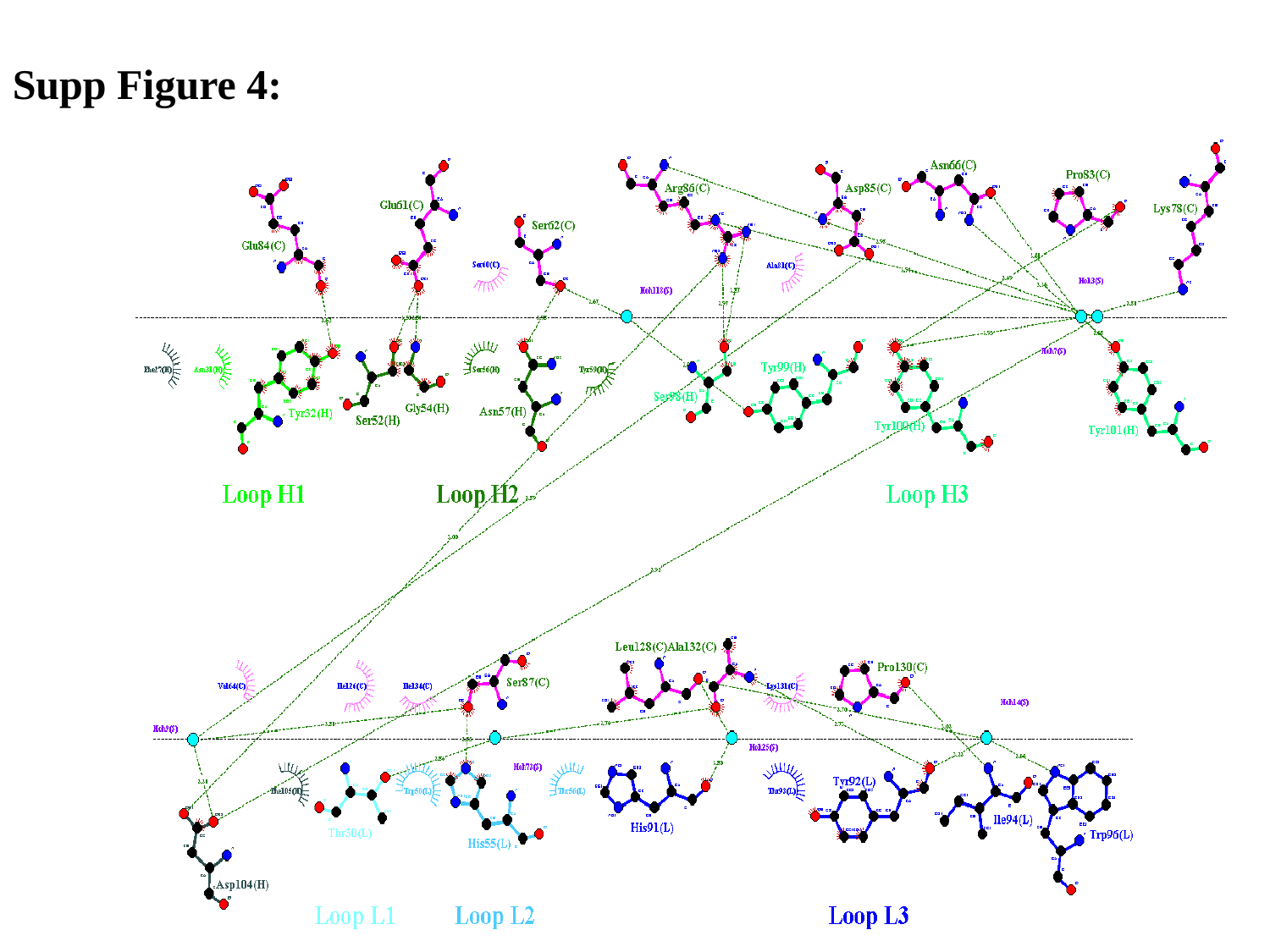

Supp Figure 4:

Supplement: S4 Fig — (PPTX) [file pone.0257972.s004.pptx]
