# Supplementary material for: Structural basis of HLX10 PD-1 receptor recognition, a promising anti-PD-1 antibody clinical candidate for cancer immunotherapy
Source: PLoS One. 2021 Dec 31;16(12):e0257972. doi: 10.1371/journal.pone.0257972 (PMC8719770; doi:10.1371/journal.pone.0257972)
Supplement: S1 File — (DOCX) [file pone.0257972.s005.docx]

**Supplemental Material and Methods**

### Cell binding by Flow Cytometry

### Binding of HLX10 to PD-1 expressing CHO cells Chinese hamster ovary (CHO) cell lines that express recombinant human PD-1 at the cell surface were developed and used to determine the specificity of PD-1 human monoclonal antibodies by flow cytometry. CHO cells were transfected with expression plasmids containing full length cDNA encoding transmembrane forms of PD-1. Binding of HLX10 and Nivo was assessed by incubating the transfected cells with the serial diluted anti-PD-1 monoclonal antibodies in FACS buffer (PBS with 1% FBS). The cells were washed with flow buffer and binding was detected with a biotin-labeled rabbit anti-human IgG Fey Ab and streptavidin-PE. Flow cytometric analyses were performed using the Cytomics FC 500 (Beckman Coulter Inc.).

***In vitro* receptor occupancy assay**

Whole blood was freshly obtained from four healthy donors. Whole blood from four healthy donors was pretreated with serial diluted HLX10 or a negative control antibody (anti-PD-L1 antibody) and incubated at 4℃ for 30 minutes. After incubation, samples underwent a red blood cell lysis step and subsequent washes with the wash buffer (DPBS+2%FBS). The leukocytes were blocked with Human Fc Receptor Binding Inhibitor (eBioscience). Biotin-conjugated competing PD-1 antibody was then added to detect free PD-1 on the cell surface. After a 30-minute incubation at 4°C, cells were stained with streptavidin-PE (eBioscience) and anti-human CD3 PE-Cyanine7 (eBioscience) or anti-human CD3/TCRE PE-Cyanine5 (Thermo Fisher Scientific) at 4°C for another 30 minutes. The CD3^+^ T cells were analyzed by Cytomic FC500 Flow Cytometer (Beckman Coulter). The receptor occupancy (RO) of HLX10 on CD3^+^ T cells was calculated using the following equation:

RO (%) = (MFI_unsaturation_-MFI_sample_)/(MFI_unsaturation_-MFI_saturation_)×100%

MFI_unsaturation_: mean fluorescence intensity of samples without HLX10 pretreatment

MFI_sample_: mean fluorescence intensity of samples pretreated with serial diluted HLX10

MFI_saturation_: mean fluorescence intensity of samples pretreated with the saturation concentration (10 μg/mL) of HLX10

**MC-38 tumor model:**

For MC-38 model, B-hPD-1 Plus mice (N=8 per group) were subcutaneously injected with MC38 tumor cells (5×10^5^) suspended in 0.1 mL PBS in the right front flank for tumor development. Tumor-bearing animals were randomly enrolled into two study groups when the mean tumor size reached 96 mm^3^. HLX10 and vehicle were intraperitoneally administrated to tumor-bearing mice at a frequency of twice per week for total six times. The tumor volume and body weight were measured and recorded twice per week.

**Surface Plasmon Resonance binding studies**

HLX10 and Nivo -PD-1 ECD interactions were analyzed by surface plasmon resonance (SPR) experiments using an SPR-2 Affinity Sensor (Sierra Sensors, Hamburg, Germany). A carboxymethylated sensor chip was pre-equilibrated with running buffer [PBS + 0.05% (v/v) Tween-20] and activated by NHS (N-hydroxysuccinimide) (Thermofisher, NY, USA) and EDC [N-ethyl-N'-(3-dimethylaminopropyl) carbodimide] (Thermofisher, NY, USA) for the immobilization of the proteins on the chip surface. After immobilization of the anti-human IgG Fc antibody (Jackson Immuno Research Laboratories Inc., PA, USA), HLX10 or Nivo were injected into flow cells for capturing by the anti-human Fc antibody. Three concentrations (58.8, 19.6 and 6.5 nM) of recombinant human PD-1 ECD were injected into both flow cells for 200 seconds and dissociation of the protein from the captured IgG on the sensor chip was allowed to proceed for 600 seconds. The apparent dissociation (k_d_) and association (k_a_) rate constants and the apparent dissociation equilibrium constant (K_D_) were calculated with the MASS-1 analysis software (Analyzer, Sierra Sensors).

**HLX10 Fragmentation**

Equal volume of HLX10 solution (2 mg/ml) and papain solution (contained 20 mM L-cysteine and 20 mM EDTA in PBS pH 6.0) were mixed and incubated at 37 °C overnight. One-tenth volume of 0.3 M iodoacetamide solution (IAA) was added to terminate the fragmentation reaction. HLX10 Fab fragment was sequentially purified by affinity protein-A, protein-L, Superdex-75, and cation ions exchange columns. The purified HLX10 Fab was dialyzed against 40 mM NaCl, 20 mM MES pH 8.5 and then concentrated to 20~25 mg/ml for crystallization.

**Preparation of recombinant human PD-1（hPD-1）**

The gene encoding the ectodomains of hPD-1 (32–160, cys 93 exchanged to serine) for crystallographic studies was cloned into pET-22b vector and expressed in *Escherichia coli* BL21(DE3) as inclusion bodies. hPD-1 was renatured by dialysis and purified as Lee’s paper described (Lee, J. Y. *et al*. Structural basis of checkpoint blockade by monoclonal antibodies in cancer immunotherapy. *Nat. Commun.* **7,** 13354 doi: 10.1038/ncomms13354 (2016).

**Crystallization of HLX10 Fab hPD-1 complex and data collection**

Purified hPD-1 and HLX10 Fab were mixed in 2:1 molar ratio, and the complex was purified by gel filtration in 20 mM Tris pH 7.0 containing 150 mM NaCl and 1mM TCEP. The complex of hPD-L1/ HLX10 Fab was concentrated to 12mg/ml. Crystallization screens at 18° were carried out by sitting-drop vapor diffusion and commercially available buffer sets were used. Diffraction-quality crystals of hPD-L1/ HLX10 Fab complex were obtained from 0.1 M Tris pH 7.5 containing 23% (w/v) PEG 4000. Crystals were flash cooled in liquid nitrogen with 25% glycerol cryoprotection. Data were collected on BL19U at SSRF.

**Structure determination and refinement**

The structure of PD-1 ectodomain with the Fab of HLX10 complex was solved by molecular replacement (MR) methods using the program Phenix (Python-based Hierarchical Environment for Integrated Xtallography). Searching models for MR were derived from the coordinates of human PD-1 ectodomain complexed with Pembrolizumab Fab (PDB: 5JXE). Separate search models for Fab were selected for the variable and constant regions basis on the sequence identity. Both molecules included PD-1 ectodomain, variable region of HLX10’s Fab in the asymmetric unit were located. Initial phases were improved by rigid body refinement, followed by rounds of simulated annealing and anisotropic B-factor refinement using the Phenix suite. The constant region of light chain of HLX10’s Fab was rebuilt manually by Coot. Coordinates of the HLX10 Fab hPD-1 complex structure have been deposited with the RCSB protein data bank (PDB id code 7E9B), Structures and protein-protein interaction were analyzed by molecular operating environment (MOE) and LigPlot+ (Laskowski R A, Swindells M B (2011). LigPlot+: multiple ligand-protein interaction diagrams for drug discovery. J. Chem. Inf. Model., 51, 2778-2786). Binding interface area was calculated by jsPISA (E. Krissinel (2015) Stock-based detection of protein oligomeric states in jsPISA, Nucl. Acids Res., DOI:10.1093/nar/gkv314).  Figures were drawn using PyMOL (The PyMOL Molecular Graphics System, Schrödinger, LLC).

***In vivo* mouse studies animal welfare:**

Mice (6-8 weeks, 20-35 g) were housed in a group of four in standard cages (D 29.5 x W 18.8 x H 13 cm). All mice were housed under a 12 hours light/dark cycle at 20±2 ℃ with unlimited sterilized food and water. Wood shavings were provided to all mice as nesting materials for environmental enrichment. Cages were changed two times per week.

Many studies have shown that analgesics can influence tumor growth and tumor microenvironment and cannot completely relieve cancer pain. In this study, we adopted early endpoint, including body weight, tumor volume, and physical conditions to monitor animal health. Detail procedures are described in the following sections:

Compressed CO_2_ gas was used for mice sacrifice. When mice reached the humane endpoints, mice were placed in the home cage and introduced 100% CO_2_ with a fill rate of 30% of the chamber volume per minute with CO_2_. The expected time to unconsciousness is usually within 2 to 3 minutes. Mice within lack of respiration and faded eye color were observed. CO_2_ flow was maintained for a minimum of 1 minute after respiration ceases. Mice death was confirmed by ascertaining cardiac and respiratory arrest or noting an animal's fixed and dilated pupils.

The maximum tumor volume was 2500 mm^3^. Tumor volume grew over 2500 mm^3^ was identified to meet the humane endpoints.

All the mice were monitored twice per week after tumor inoculation. Bodyweight (BW), tumor volume, and general physical conditions were observed and recorded. Weight loss over 20% of initial BW or tumor volume grew over 2000 mm^3^ were identified to meet the humane endpoints that mice should be euthanized with approval protocol.

**Non-Human Primate Monkey Studies:**

Pharmacokinetic study of HLX10 were performed at JOININ Laboratories (Beijing Economic-Technological Development) testing facility in China. The studies were conducted in accordance with the protocol and applicable JOININ’s Standard Operating Procedures (SOPs). Animal care was compliant with the relevant JOINN’s SOPs, the Guide for the Care and Use of Laboratory Animals, 8th Edition (Institute of Laboratory Animal Resources, Commission on Life Sciences, National Research Council; National Academy Press; Washington, D.C., 2010), and the U.S. Department of Agriculture (USDA) through the Animal Welfare Act (Public Law 99-198). Animals in a group per sex were housed in stainless steel cages and in an environmentally monitored, well-ventilated room (conventional grade) maintained at a temperature of 18 - 26°C and a relative humidity of 40 to 70%. Fluorescent lighting provided illumination approximately a 12 hours light/dark cycle per day. Certified commercial monkey maintenance diet was provided to each monkey at approximately 200 g/day twice daily and fruit approximately 50 g/day once daily. The diet met the State Standard of the People’s Republic of China GB14924.2-2001 and GB14924.3-2010. Tap water was provided ad libitum during the quarantine and study periods. Samples of water from the animal facility were analyzed annually for toxicological parameters (heavy metals), and monthly for appearances and microbiological parameters. The water conformed to the drinking water standards according to the State Standard of the People’s Republic of China GB14925-2010. Monkeys were observed at least twice daily (am and pm) during the acclimation and study periods for clinical signs which included, but not limited to mortality, morbidity, respiration, secretion, feces, emesis and capability of water and food intake. After the last PK and RO sampling, all animals were returned to the stock colony of the animal facility department.
