# Supplementary material for: Structural basis of HLX10 PD-1 receptor recognition, a promising anti-PD-1 antibody clinical candidate for cancer immunotherapy
Source: PLoS One. 2021 Dec 31;16(12):e0257972. doi: 10.1371/journal.pone.0257972 (PMC8719770; doi:10.1371/journal.pone.0257972)
Supplement: S2 File — (DOCX) [file pone.0257972.s006.docx]

Non-Human Primate Monkey Studies:

Pharmacokinetic study of HLX10 were performed at JOININ Laboratories (Beijing Economic-Technological Development) testing facility in China. The studies were conducted in accordance with the protocol and applicable JOININ’s Standard Operating Procedures (SOPs). Animal care was compliant with the relevant JOINN’s SOPs, the Guide for the Care and Use of Laboratory Animals, 8th Edition (Institute of Laboratory Animal Resources, Commission on Life Sciences, National Research Council; National Academy Press; Washington, D.C., 2010), and the U.S. Department of Agriculture (USDA) through the Animal Welfare Act (Public Law 99-198). Animals in a group per sex were housed in stainless steel cages and in an environmentally monitored, well-ventilated room (conventional grade) maintained at a temperature of 18 - 26°C and a relative humidity of 40 to 70%. Fluorescent lighting provided illumination approximately a 12 hours light/dark cycle per day. Certified commercial monkey maintenance diet was provided to each monkey at approximately 200 g/day twice daily and fruit approximately 50 g/day once daily. The diet met the State Standard of the People’s Republic of China GB14924.2-2001 and GB14924.3-2010. Tap water was provided ad libitum during the quarantine and study periods. Samples of water from the animal facility were analyzed annually for toxicological parameters (heavy metals), and monthly for appearances and microbiological parameters. The water conformed to the drinking water standards according to the State Standard of the People’s Republic of China GB14925-2010. Monkeys were observed at least twice daily (am and pm) during the acclimation and study periods for clinical signs which included, but not limited to mortality, morbidity, respiration, secretion, feces, emesis and capability of water and food intake. After the last PK and RO sampling, all animals were returned to the stock colony of the animal facility department.
