# Supplementary material for: Structural basis of HLX10 PD-1 receptor recognition, a promising anti-PD-1 antibody clinical candidate for cancer immunotherapy
Source: PLoS One. 2021 Dec 31;16(12):e0257972. doi: 10.1371/journal.pone.0257972 (PMC8719770; doi:10.1371/journal.pone.0257972)
Supplement: Attachment — Submitted filename: Response to Reviewers.docx [file pone.0257972.s007.docx]

Re-rebuttal letter:

Dear Editor,

We thank the reviewers for their insightful comments on the manuscript and have edited the manuscript to address their concerns. We submitted 2 versions: final version and one with track changes.

We believe that the manuscript is now suitable for publication in PLOS.

We also modified some items in the manuscript to satisfy the editorial guidelines. The followings are the answers to the reviewer’s comments/questions and the list of edits made.

Reviewer #1: The authors present an extensive study describing the generation of an anti-PD-1 IgG4 monoclonal antibody with a remarkable activity in vitro (binding to PD-1, to PD-1 expressing cells, inhibition of PD-1/PD-L1 binding, activity on humanT cells) and in vivo in several tumor models. Also a pharmacokinetic study in cynomolgus monkeys has been performed is here reported. The antibody named HLX10 appears as a promising new candidate for immunotherapy.
The work is well designed, well executed and well presented and can be accepted with minor revisions.
A more detailed description of the phage display library construction and screening should be provided in terms of complexity, panning rounds, number of new sequences etc.

Thank you for the comments. We have added more details about the phage library construction, and screening etc. to the material and method section.

Reviewer #2: In this paper, author reported that HLX10, a novel fully humanized anti PD-1 IgG4 mAb, blocked PD1/PD-L1 interaction and activated T-cell proliferation and cytokine secretion in vitro. HLX10 demonstrated significant antitumor efficacy in several in 5 mouse models and synergized with Avastin biosimilar to promote robust tumor activity. Finally, author showed a co-crystal structure of the antigen-binding fragment (Fab) of HLX10 in complex with PD-1 at a 1.78-Å resolution. Besides, HLX10 was well tolerated by Monkey single dose pharmacokinetics assay. Altogether, comprehensive study proved that HLX10 had better bioactivity to FDA approved nivolumab and pembrolizumab.

Minor questions:

1. As author described “The EMT-6 is considered an anti-PD-1 resistant tumor, due to an immune excluded phenotype, macrophage-derived suppressive infiltration, and epithelial-to-mesenchymal transition (EMT) [22, 23]. Upon treatment of established EMT-6/ tumors in BALB/c mice, HLX10 was able to significantly inhibit tumor growth with some responding and nonresponding mice. Please add a short discussion. You may refer recent study on anti-PD-L1/TGF-b bispecific antibody (Yi M, et al. The construction, expression, and enhanced anti-tumor activity of YM101: a bispecific antibody simultaneously targeting TGF-β and PD-L1. J Hematol Oncol, 2021;14:27).
2. HLX10 was shown to synergize with Avastin biosimilar to promote robust tumor activity. For background information as to the combination of ICI with anti-angiogenesis, please refer recent review and make short discussion (Yi M, et al. Synergistic effect of immune checkpoint blockade and anti-angiogenesis in cancer treatment. Mol Cancer, 2019; 19:60).
3. Could you please show CD8+ T-cells and blood vessel for tumor tissues from in vivo study of Fig 8A, B?

Thank you for the comments and questions. We have added shorth discussions to the results section, and we have added the references suggested.

We agree elucidating the synergy between ICI and anti-angiogenesis inhibitors is an important question and would have been nice to show during these studies. We referenced (Yi M, et al. Synergistic effect of immune checkpoint blockade and anti-angiogenesis in cancer treatment. Mol Cancer, 2019; 19:60) review, which will add some background and general context.

Unfortunately, CD8+ and blood vessel were not examined in these studies as they were not the primary goals of the experiment.

Reviewer #3: The research article by Hassan Issafras, Shilong Fan et al. deals with the structural, biological and pharmacological characterizations of HLX10, a humanized IgG4 monoclonal antibody against PD-1 receptor.
A comparative analysis of binding activity of HLX10 and several approved mAb to PD-1 was carried out, showing a similar efficiency of HLX10 to the reference antibody Nivolumab in enhancing T-cell responses and cytokine production in vitro. The anticancer activity of HLX10 was then demonstrated using several syngeneic and xenograft models. Moreover, an increase of antitumor efficacy of HLX10 was observed when used in combination with HLX10 enhanced an anti-angiogenic antibody (HLX04).
Crystallographic studies of HLX10/PD-1 complex disclose a similar binding mode to the approved Pembrolizumab mAb and identify Arg86 of PD-1 as key residue for HLX10 binding. PK studies in cynomolgus monkey showed that HLX10 is endowed with a small volume of distribution and long elimination half-life, which is common to recombinant monoclonal antibodies. Toxicity studies show also that HLX10 is well tolerated up to 100 mg/kg for 4 weeks. Overall, the work is well conceived, the English language is very good, and results are of interest to researchers working in the field of cancer immunotherapies. The methodological part and experiments are well executed and compliant to a high technical standard. Conclusions are appropriately written and supported by the results.
However, before accepting the paper for publication in PLOS One, authors should address the following minor issues.


- Line 303-305: Authors should mention the technique used to evaluate HLX10 binding to PD-1 in CHO transfected cells (in the same way as it is reported for the BLI assay to check the monomeric binding affinity).

The flow cytometry method used to evaluate HLX10 binding to PD-1 in CHO transfected cells is described in supplemental MM

- Line 412: The text ‘Error! Reference source not found’ appears, the author should insert the citation properly.

Error corrected

- Line 428: Authors should mention that crystallographic methods are reported in supplementary Material

Added

- The legend and clarity of figure 6 should be improved. It is indeed unclear the meaning of the nine
growth curves (solid black lines) representing HLX10 15 mg/kg. Do they show the growth of EMT breast cancer syngeneic tumors following the injections (6 or 9 injections) ?

Each black curve represents an individual animal (N=10). The dotted line represents the average of 10 animals from the vehicle group. We have added additional description to the legend to make the figures clearer. The shaded area is the treatment window (6 injections). We hope this will be satisfactory.

- In the legend of figure 9 it is stated that “Arg86 of hPD-1 is involved in several hydrogen bonds, salt bridge and hydrophobic interaction with HLX10.” Being a polar positively charged residue, Arg86 cannot form hydrophobic interactions with HLX10.

We corrected this error.

- In the conclusion part, authors should specify that the combination therapy HLX10/HLX04 (NCT03757936) is a phase I clinical study.

We have added this info

1. Please ensure that your manuscript meets PLOS ONE's style requirements, including those for file naming. The PLOS ONE style templates can be found at

<https://journals.plos.org/plosone/s/file?id=wjVg/PLOSOne_formatting_sample_main_body.pdf> and

https://journals.plos.org/plosone/s/file?id=ba62/PLOSOne_formatting_sample_title_authors_affiliations.pdf

 checked

2.  At this time, we request that you  please report additional details in your Methods section regarding animal care, as per our editorial guidelines: 1) Please provide details of animal welfare (e.g., shelter, food, water, environmental enrichment) 2) Please describe any steps taken to minimize animal suffering and distress, such as by administering anesthetics or analgesics, 3) Please include the method of sacrifice, 4) Please include the maximum sizes tumors grew to in your experiments and 5) Please describe the post-operative and post tumor implantation care received by the animals, including the frequency of monitoring and the criteria used to assess animal health and well-being. Thank you for your attention to these requests.

The following details were added to supplemental materials and methods

 1) Please provide details of animal welfare (e.g., shelter, food, water, environmental enrichment)

Mice (6-8 weeks, 20-35 g) were housed in a group of four in standard cages (D 29.5 x W 18.8 x H 13 cm). All mice were housed under a 12 hour light/dark cycle at 20±2 ℃ with unlimited sterilized food and water. Wood shavings were provided to all mice as nesting materials for environmental enrichment. Cages were changed two times per week.

2) Please describe any steps taken to minimize animal suffering and distress, such as by administering anesthetics or analgesics

Many studies have shown that analgesics can influence tumor growth and tumor microenvironment and cannot completely relieve cancer pain. In this study, we adopted early endpoint, including body weight, tumor volume, and physical conditions to monitor animal health. Detail procedures are described in the following sections.

3) Please include the method of sacrifice

Compressed CO_2_ gas was used for mice sacrifice. When mice reached the humane endpoints, mice were placed in the home cage and introduced 100% CO_2_ with a fill rate of 30% of the chamber volume per minute with CO_2_. The expected time to unconsciousness is usually within 2 to 3 minutes. Mice within lack of respiration and faded eye color were observed. CO_2_ flow was maintained for a minimum of 1 minute after respiration ceases. Mice death was confirmed by ascertaining cardiac and respiratory arrest or noting an animal's fixed and dilated pupils.

4) Please include the maximum sizes tumors grew to in your experiments

The maximum tumor volume was 2000 mm^3^. Tumor volume grew over 2500 mm^3^ was identified to meet the humane endpoints.

5) Please describe the post-operative and post tumor implantation care received by the animals, including the frequency of monitoring and the criteria used to assess animal health and well-being.

All the mice were monitored twice per week after tumor inoculation. Bodyweight (BW), tumor volume, and general physical conditions were observed and recorded. Weight loss over 20% of initial BW or tumor volume grew over 2500 mm^3^ were identified to meet the humane endpoints that mice should be euthanized with approval protocol.

3. Thank you for stating the following in the Financial Disclosure section:

"The author(s) received no specific funding for this work.
Hassan Issafras, Chi-Ling Tseng, Yunchih Cheng, Peihua Lin, Lisa Xiao, Yun-Ju Huang, Chih-Hsiang Tu, Ya-Chin Hsiao,  Yen-Hsiao Chen, Chien-Hsin Ho, Ou Li, Yanling Wang, Sandra Chen, Zhenyu Ji, Eric Zhang, Yi-Ting Mao, Eugen Liu, Shumin Yang and Weidong Jiang were employees of either Hengenix Inc or Shanghai Henlius Biotech, Inc., P. R. China.

Shilong Fan and Min Li received funding from Shanghai Henlius Biotech, Inc."

We note that one or more of the authors have an affiliation to the commercial funders of this research study : Hengenix Inc., Shanghai Henlius Biotech, Inc., HanchorBio Inc., Ltd, Anwita Biosciences.

3.1. Please provide an amended Funding Statement declaring this commercial affiliation, as well as a statement regarding the Role of Funders in your study. If the funding organization did not play a role in the study design, data collection and analysis, decision to publish, or preparation of the manuscript and only provided financial support in the form of authors' salaries and/or research materials, please review your statements relating to the author contributions, and ensure you have specifically and accurately indicated the role(s) that these authors had in your study. You can update author roles in the Author Contributions section of the online submission form.

We have added the following statement to the manuscript and updated the authors contribution online

At the time of these analyses, authors [HI, CT, YC, PL, LX, YH, CT, YH, YC, CH, OL, YW, SC, ZJ, EZ, YM, EL, SY and WJ] were employees of either Shanghai Henlius Biotech, Inc or its subsidiary Hengenix Inc (USA). This does not alter the authors adherence to PLOS ONE policies on sharing data and materials.

Please also include the following statement within your amended Funding Statement.

See above. We also added the specific role of each author

“The funder provided support in the form of salaries for authors [HI, CT, YC, PL, LX, YH, CT, YH, YC, CH, OL, YW, SC, ZJ, EZ, YM, EL, SY and WJ], but did not have any additional role in the study design, data collection and analysis, decision to publish, or preparation of the manuscript. The specific roles of these authors are articulated in the ‘author contributions’ section.”

If your commercial affiliation did play a role in your study, please state and explain this role within your updated Funding Statement.

 Commercial affiliation did not play a role

3.2. Please also provide an updated Competing Interests Statement declaring this commercial affiliation along with any other relevant declarations relating to employment, consultancy, patents, products in development, or marketed products, etc.

Within your Competing Interests Statement, please confirm that this commercial affiliation does not alter your adherence to all PLOS ONE policies on sharing data and materials by including the following statement: "This does not alter our adherence to  PLOS ONE policies on sharing data and materials.” (as detailed online in our guide for authors http://journals.plos.org/plosone/s/competing-interests). If this adherence statement is not accurate and  there are restrictions on sharing of data and/or materials, please state these. Please note that we cannot proceed with consideration of your article until this information has been declared.

We have added this statement to the Competing Interests Statement in the manuscript

Please include both an updated Funding Statement and Competing Interests Statement in your cover letter. We will change the online submission form on your behalf.

We have added an updated funding statement to the cover letter.

Please know it is PLOS ONE policy for corresponding authors to declare, on behalf of all authors, all potential competing interests for the purposes of transparency. PLOS defines a competing interest as anything that interferes with, or could reasonably be perceived as interfering with, the full and objective presentation, peer review, editorial decision-making, or publication of research or non-research articles submitted to one of the journals. Competing interests can be financial or non-financial, professional, or personal. Competing interests can arise in relationship to an organization or another person. Please follow this link to our website for more details on competing interests: http://journals.plos.org/plosone/s/competing-interests

4. We note that you have indicated that data from this study are available upon request. PLOS only allows data to be available upon request if there are legal or ethical restrictions on sharing data publicly. For information on unacceptable data access restrictions, please see <http://journals.plos.org/plosone/s/data-availability#loc-unacceptable-data-access-restrictions>.

We corrected this statement to available online. The PDB structure and coordinates will be public before manuscript publication

In your revised cover letter, please address the following prompts:

a) If there are ethical or legal restrictions on sharing a de-identified data set, please explain them in detail (e.g., data contain potentially identifying or sensitive patient information) and who has imposed them (e.g., an ethics committee). Please also provide contact information for a data access committee, ethics committee, or other institutional body to which data requests may be sent.

 We have added a statement to the cover letter

b) If there are no restrictions, please upload the minimal anonymized data set necessary to replicate your study findings as either Supporting Information files or to a stable, public repository and provide us with the relevant URLs, DOIs, or accession numbers. Please see <http://www.bmj.com/content/340/bmj.c181.long> for guidelines on how to de-identify and prepare clinical data for publication. For a list of acceptable repositories, please see <http://journals.plos.org/plosone/s/data-availability#loc-recommended-repositories>.

We will update your Data Availability statement on your behalf to reflect the information you provide.

5. PLOS requires an ORCID iD for the corresponding author in Editorial Manager on papers submitted after December 6th, 2016. Please ensure that you have an ORCID iD and that it is validated in Editorial Manager. To do this, go to ‘Update my Information’ (in the upper left-hand corner of the main menu), and click on the Fetch/Validate link next to the ORCID field. This will take you to the ORCID site and allow you to create a new iD or authenticate a pre-existing iD in Editorial Manager. Please see the following video for instructions on linking an ORCID iD to your Editorial Manager account: <https://www.youtube.com/watch?v=_xcclfuvtxQ>

 We have added the ORCID info.

7. Please amend the manuscript submission data (via Edit Submission) to include author Chi-Ling Tseng, Yunchih Cheng, Peihua Lin, Lisa Xiao, Yun-Ju Huang, Chih-Hsiang Tu, Ya-Chin Hsiao, Min Li, Yen-Hsiao Chen3, Chien-Hsin Ho, Ou Li, Yanling Wang, Sandra Chen, Zhenyu Ji, Eric Zhang, Yi-Ting Mao, Eugen Liu, Shumin Yang.

 We have amended the manuscript submission data.

8. We note that you have included the phrase “data not shown” in your manuscript. Unfortunately, this does not meet our data sharing requirements. PLOS does not permit references to inaccessible data. We require that authors provide all relevant data within the paper, Supporting Information files, or in an acceptable, public repository. Please add a citation to support this phrase or upload the data that corresponds with these findings to a stable repository (such as Figshare or Dryad) and provide and URLs, DOIs, or accession numbers that may be used to access these data. Or, if the data are not a core part of the research being presented in your study, we ask that you remove the phrase that refers to these data.

 We either removed “data not shown” or added supporting data in supplementals

9. Please include captions for your Supporting Information files at the end of your manuscript, and update any in-text citations to match accordingly. Please see our Supporting Information guidelines for more information: http://journals.plos.org/plosone/s/supporting-information.

  We added a caption of supporting information as suggested
